# Oxymatrine inhibits the proliferation of prostate cancer cells *in vitro* and *in vivo*

**DOI:** 10.3892/mmr.2015.3338

**Published:** 2015-02-12

**Authors:** CUNZAO WU, WEIPING HUANG, YONG GUO, PENG XIA, XIANBIN SUN, XIAODONG PAN, WEILIE HU

**Affiliations:** 1 Graduate School, Southern Medical University, Guangzhou, Guangdong 510515, P.R. China; 2 Transplantation Center, The First Affiliated Hospital, Wenzhou Medical University, Wenzhou, Zhejiang 325013, P.R. China; 3 Department of Urology, Guangzhou General Hospital of Guangzhou Military Command, Guangzhou, Guangdong 510010, P.R. China

**Keywords:** prostate cancer, oxymatrine, proliferation, apoptosis

## Abstract

Oxymatrine is an alkaloid, which is derived from the traditional Chinese herb, *Sophora flavescens* Aiton. Oxymatrine has been shown to exhibit anti-inflammatory, antiviral, and anticancer properties. The present study aimed to investigate the anticancer effects of oxymatrine in human prostate cancer cells, and the underlying molecular mechanisms of these effects. An MTT assay demonstrated that oxymatrine significantly inhibited the proliferation of prostate cancer cells in a time- and dose-dependent manner. In addition, flow cytometry and a terminal deoxynucleotidyl transferase-mediated dUTP-biotin nick end-labeling assay suggested that oxymatrine treatment may induce prostate cancer cell apoptosis in a dose-dependent manner. Furthermore, western blot analysis demonstrated a significant increase in the expression of p53 and bax, and a significant decrease in that of Bcl-2, in prostrate cancer cells in a dose-dependent manner. *In vivo* analysis demonstrated that oxymatrine inhibited tumor growth following subcutaneous inoculation of prostate cancer cells into nude mice. The results of the present study suggested that the antitumor properties of oxymatrine, may be associated with the inhibition of cell proliferation, and induction of apoptosis, via the regulation of apoptosis-associated gene expression. Therefore, the results may provide a novel approach for the development of prostate cancer therapy using oxymatrine, which is derived from the traditional Chinese herb, *Sophora flavescens*.

## Introduction

Prostate cancer is the second most common type of cancer and the sixth leading cause of cancer-related mortality among males worldwide (1). Currently, prostate-specific antigen testing, digital rectal examination and histopathological evaluation of prostate needle biopsies, are performed in order to detect and monitor the progression of prostate cancer (2,3). However, there is increasing evidence to suggest that the resistance of prostate cancer cells to conventional drugs is a significant problem. The investigation of potential novel and effective drugs for the treatment of prostate cancer is therefore required (4).

Traditional Chinese herbs are sources of compounds that may serve as potential therapeutic drugs for cancer (5). Ku Shen, the dried root of *Sophora flavescens* Aiton, is a commonly used, traditional Chinese herbal medicine. Oxymatrine, an alkaloid present in Ku Shen, exhibits anti-inflammatory, anti-allergic, antiviral, antifibrotic and cardiovascular-protective properties (6–8). Furthermore, oxymatrine has been reported to exhibit anticancer properties, such as the inhibition of cancer cell proliferation, the cell cycle and angiogenesis, the promotion of cell apoptosis and reversal of multi-drug resistance in patients with cancer (9–11). A previous study suggested that oxymatrine may suppress angiogenesis by modulating the expression of the NF-κB-mediated vascular endothelial growth factor signaling pathway (12). Furthermore, oxymatrine may induce mitochondria-dependent apoptosis in human osteosarcoma cells by inhibiting the phosphatidylinositol-3 kinase/protein kinase B pathway (13). A number of studies have demonstrated that oxymatrine may inhibit cell growth and the cell cycle, and promote apoptosis in human gastric and breast cancers (9,14,15). However, to the best of our knowledge, the effects of oxymatrine on prostate cancer cells have yet to be investigated. Therefore, the present study aimed to investigate the anticancer effects of oxymatrine on human prostate cancer cells.

## Materials and methods

### Reagents

Dulbecco’s modified Eagle’s medium (DMEM), fetal bovine serum (FBS) and antibiotics (penicillin and streptomycin) were purchased from Invitrogen Life Technologies (Carlsbad, CA, USA). Oxymatrine, obtained from Sigma-Aldrich (St. Louis, MO, USA), was dissolved in dimethyl sulfoxide (Sigma-Aldrich) with the stock concentration of 10 mg/ml, and further diluted in the culture medium. Each experiment was repeated at least three times and new dilutions were prepared for each experiment.

### Cell culture

DU145 and PC-3 human prostate cancer cell lines and the PNT1B healthy human prostate cell line were obtained from the Chinese Academy of Sciences (Shanghai, China). Cell lines were cultured in DMEM supplemented with 10% FBS, 100 IU/ml penicillin and 100 mg/ml streptomycin, and incubated at 37°C in 5% CO_2_ for 48 h.

### Cell proliferation assay

DU145, PC-3 and PNT1B cell lines were seeded into 96-well plates, incubated overnight and treated with oxymatrine (0, 2, 4, 6 and 8 mg/ml). Cell viability was determined using an MTT assay (Sigma-Aldrich). Cells (3×10^4^ cells/well) were seeded into 96-well plates and incubated overnight at 37°C in 5% CO_2_. Subsequently, the cells were incubated with different concentrations of oxymatrine (0, 2, 4, 6 and 8 mg/ml). MTT (10 ml; 5 mg/ml) was added and the mixture was incubated in darkness at 37°C for 2 h. Absorbance was measured at a wavelength of 490 nm using a microplate reader (FluoDia T70; Photon Technology International, Lawrenceville, NJ, USA).

### Flow cytometric analysis

Human prostate cancer cell lines were treated with different concentrations of oxymatrine (0, 4 and 8 mg/ml). Following treatment with oxymatrine for 48 h, cells were trypsinized (Sigma-Aldrich) and centrifuged at 1,000 x g and the pellet was washed twice using PBS. Cells were resuspended and washed with PBS three times. Apoptotic cells were detected using an annexin V-fluorescein isothiocyanate/propidium iodide (annexin V-FITC/IP) cell apoptosis detection kit, according to the manufacturer’s instructions (BD Biosciences, Franklin Lakes, NJ, USA).

### Western blot analysis

Following oxymatrine treatment, proteins were extracted and separated using a sodium dodecyl sulfate polyacrylamide electrophoresis gel (Bio-Rad Laboratories, Inc., Hercules, CA, USA). Proteins were then transferred to polyvinylidene difluoride membranes (EMD Millipore, Billerica, MA, USA). Membranes were blocked and incubated with the following primary antibodies: Mouse anti-human p53 monoclonal antibody (1:1,000 dilution; cat. no. sc-126), mouse anti-human bcl-2 monoclonal antibody (1:1,000 dilution; cat. no. sc-7382), mouse anti-human bax monoclonal antibody (1:1,000 dilution; cat. no. sc-20067) and mouse anti-human GAPDH monoclonal antibody (1:5,000 dilution; cat. no. sc-365062) (Santa Cruz Biotechnology, Inc., Dallas, TX, USA) overnight at 4°C. Following washing with Tris-buffered saline and Tween, membranes were incubated with a goat anti-mouse secondary antibody conjugated with horseradish peroxidase (1:10,000 dilution; cat. no. sc-2072; Santa Cruz Biotechnology, Inc.) and visualized using an enhanced chemiluminescent detection reagent from Pierce Biotechnology, Inc. (Rockford, IL, USA).

### In vivo xenografts

Approval was obtained from the ethics committee of the First Affiliated Hospital of Wenzhou Medical University (Wenzhou, China). BALB/c homozygous (nu/nu) nude mice (aged 6–8, weeks; weight, 18–20 g), bred in-house, were maintained in a specific pathogen-free environment. PC-3 cells (3×10^6^) were suspended in 100 μl PBS and subcutaneously injected into the left axilla of recipient mice. On day five, 24 tumor-bearing mice were randomly divided into three groups: The control group was treated with PBS, and two groups were treated with different concentrations of oxymatrine (50 mg/kg and 100 mg/kg body weight). Oxymatrine was administered to the mice, using daily intraperitoneal injections. Tumor volume was calculated using the formula A × B^2^ × π/6, where A was the length of the longest aspect of the tumor, and B was the length of the tumor perpendicular to A. Following five weeks of treatment the mice were sacrificed by cervical dislocation and tumor weight was measured.

### A terminal deoxynucleotidyl transferase-mediated dUTP-biotin nick end-labeling (TUNEL) assay

Cell apoptosis in mouse tumor samples from six BALB/c mice, was measured *in vivo*, using a TUNEL assay kit (Roche diagnostics, Indianapolis, IN, USA). Brown nuclei were considered apoptotic. The number of apoptotic cells/1,000 cells was recorded in each field of view, using a microscope (LZ12; Leica Microsystems GmbH, Wetzlar, Germany) at magnification ×200.

### Statistical analysis

Data are expressed as the mean ± standard deviation and statistical analysis was carried out using SPSS version 10.0 (SPSS, Inc., Chicago, IL, USA). Comparisons between groups were made using analysis of variance. P<0.05 was considered to indicate a statistically significant difference.

## Results

### Oxymatrine inhibits the proliferation of prostate cancer cells

**Figure 1:**
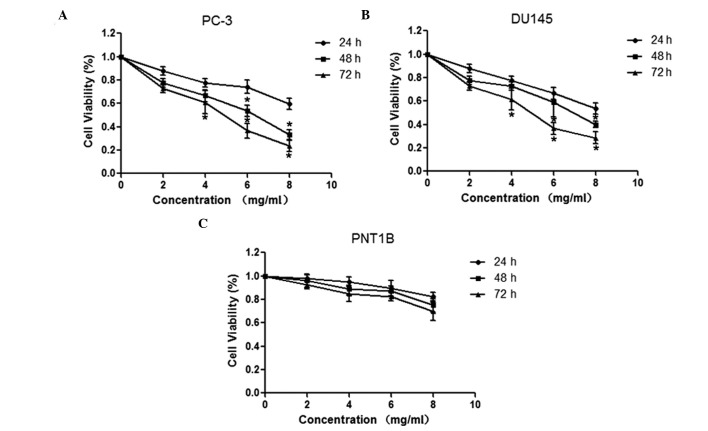
Oxymatrine inhibited the proliferation of prostate cancer cells *in vitro*. (A) PC-3 and (B) DU145 prostate cancer cells, and (C) PNT1B healthy prostate cells were treated with different concentrations of oxymatrine for 24, 48 and 72 h. Cell proliferation was assessed using an MTT assay. Values represent the mean ± standard deviation of three independent experiments. ^*^P<0.05.

In order to investigate the antiproliferative effects of oxymatrine on prostate cancer cells, DU145 and PNT1B cell lines were treated with different concentrations of oxymatrine (0, 2, 4, 6 and 8 mg/ml) for 24, 48 and 72 h. An MTT assay suggested that oxymatrine significantly inhibited the proliferation of DU145 and PC-3 cell lines in a time- and dose-dependent manner (Fig. 1A and B). By contrast, following treatment with oxymatrine, PNT1B healthy human prostate cell proliferation was not inhibited (Fig. 1C).

### Oxymatrine promotes prostate cancer cell apoptosis

**Figure 2:**
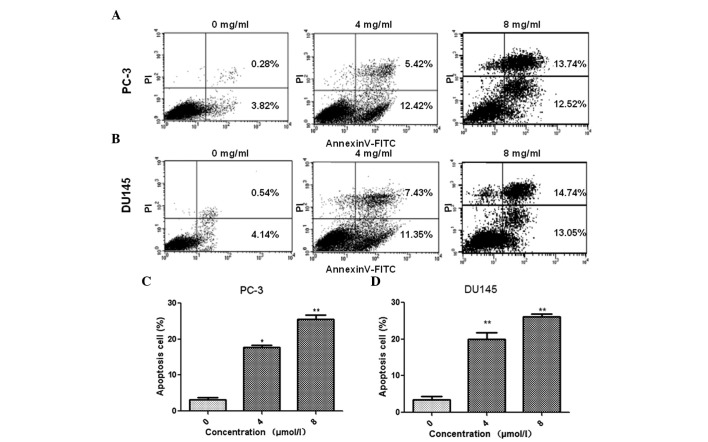
Oxymatrine promoted prostate cancer cell apoptosis. (A) and (C) PC-3, and (B) and (D) DU145 cells were treated with different concentrations of oxymatrine for 48 h. Annexin V-fluorescein isothiocyanate/propidium iodide staining was conducted in order to measure cell apoptosis. Values represent the mean ± standard deviation of three independent experiments. ^*^P<0.05 and ^**^P<0.01.

Oxymatrine-induced apoptosis in prostate cancer cells was measured using annexin V-FITC/PI double staining. Flow cytometry analysis demonstrated that treatment with oxymatrine resulted in a significant increase in cell apoptosis of PC-3 (Fig. 2A and C) and DU145 (Fig. 2B and D) cell lines, in a dose-dependent manner. These data suggested that oxymatrine treatment may promote prostate cancer cell apoptosis.

### Effect of oxymatrine on the expression of apoptosis-related proteins

**Figure 3:**
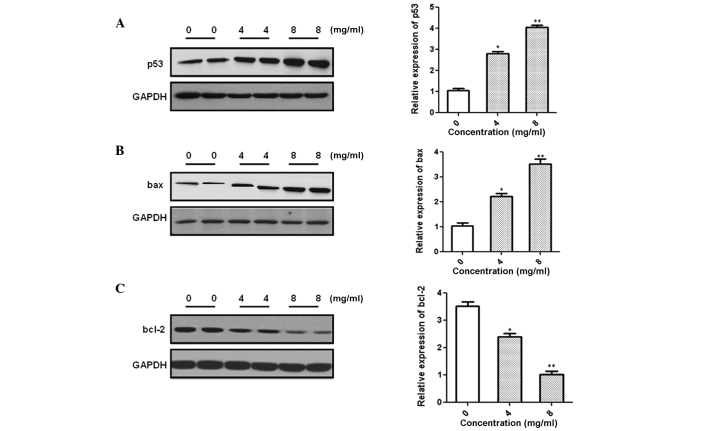
Effect of oxymatrine treatment on apoptosis-associated protein expression in prostate cancer cells. Following treatment with oxymatrine for 48 h, cell lysates were prepared and western blot analysis was performed in order to measure the expression of p53, bax and bcl-2. GAPDH was used as a positive control. Results of representative blots are provided. Relative band intensities were used in order to quantify (A) p53, (B) bax, and (C) bcl-2 protein expression levels. ^*^P<0.05 and ^**^P<0.01.

In order to investigate the possible molecular mechanisms underlying oxymatrine-induced apoptosis of prostate cancer cells, the expression of p53, bax and bcl-2 was analyzed following treatment with different concentrations of oxymatrine. Western blotting suggested that the expression of p53 and bcl-2 decreased, whereas that of bax increased, in a dose-dependent manner (Fig. 3).

### Oxymatrine reduces prostate cancer cell proliferation in vivo

**Figure 4:**
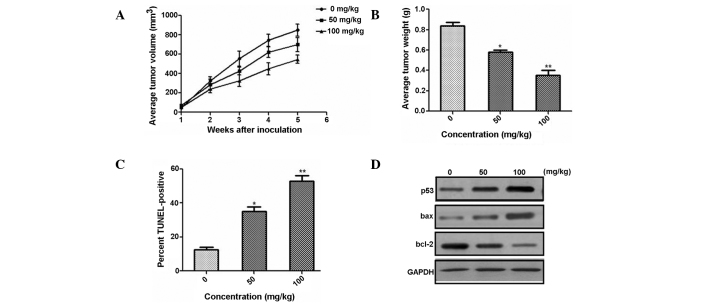
Oxymatrine inhibited PC-3 cell proliferation *in vivo*. Three concentration levels of oxymatrine (0, 50 and 100 ml/kg) were intraperitoneally injected into nude mice using PC-3 subcutaneous xenografts. (A) Tumor volume and (B) weight of subcutaneous xenografts were measured at one week intervals. Cell apoptosis in tumor tissues was measured using (C) TUNEL and (D) western blotting of apoptosis-associated proteins, p53, bax and bcl-2. ^*^P<0.05 and ^**^P<0.01. TUNEL, terminal deoxynucleotidyl transferase-mediated dUTP-biotin nick end-labeling.

In order to investigate the effect of oxymatrine on tumor growth *in vivo*, three concentration levels of oxymatrine were intraperitoneally injected into nude mice, using PC-3 subcutaneous xenografts. The results suggested that the volume (Fig. 4A) and weight (Fig. 4B) of tumors in mice significantly decreased in a dose-dependent manner. A TUNEL assay suggested that the number of apoptotic cells increased significantly in a dose-dependent manner (Fig. 4C). In accordance with the *in vitro* analyses, the expression of apoptosis-associated proteins, p53 and bcl-2 decreased and that of bax increased, in a dose-dependent manner (Fig. 4D). Oxymatrine may therefore reduce prostate cancer cell growth by promoting cell apoptosis *in vivo*.

## Discussion

Oxymatrine is an alkaloid, which is derived from Ku Shen and has been shown to be a potential treatment for a number of types of cancers, such as pancreatic (11), gastric (14) and breast cancer (15). However, to the best of our knowledge, the effects of oxymatrine on prostate cancer and the underlying molecular mechanisms of these effects have yet to be investigated. In the present study, oxymatrine treatment was found to promote prostate cancer cell apoptosis and inhibit prostate cancer cell proliferation *in vitro* and *in vivo*.

*In vitro*, an MTT assay demonstrated that oxymatrine treatment significantly inhibited cell proliferation in DU145 and PC-3 prostrate cancer cell lines in a time- and dose-dependent manner. Xenograft tumorigenesis analysis *in vivo*, demonstrated that following oxymatrine treatment, the weight and size of tumors in PC-3 subcutaneous xenografts were significantly reduced, in a dose-dependent manner. The results of the present study, therefore, indicated that oxymatrine treatment inhibited the proliferation of prostate cancer cells, *in vitro* and *in vivo*.

Apoptosis is the process of cell death, characterized by cellular and molecular processes, such as phosphatidylserine externalization, cell shrinkage and chromatin condensation (16,17). Uncontrolled cell proliferation is involved in tumor initiation and progression. Therefore, apoptosis induction provides a potential mechanism for the development of antitumor therapies (18,19). In the present study, oxymatrine treatment induced prostate cancer cell apoptosis *in vitro*, in a dose-dependent manner, which was demonstrated using flow cytometry and TUNEL analysis.

A number of signalling pathways are involved in the regulation of apoptosis, and numerous molecular markers involved in these pathways have been identified (20–22). For example, p53, encoded by the tumor protein 53 gene is associated with cell apoptosis and cell cycle regulation, in multi-cellular organisms (21,22). Upon internal and external stimuli, such as oxidative stress and viral infection, p53 may activate or suppress a number of downstream target genes involved in apoptosis, such as bax, p53 upregulated modulator of apoptosis and bcl-2 (23,24). Bax is a p53 primary-response gene, involved in a p53-regulated pathway. p53 accumulates in the cytosol and promotes the expression of bax, which permeabilizes mitochondria and promotes cell apoptosis (25,26). The antiapoptotic protein, bcl-2 has been shown to prevent mitochondrial disruption and block cytochrome *c* release from the mitochondria (27,28). The results of the present study demonstrated that oxymatrine treatment of prostate cancer cells may result in an increase in p53 and bax expression and a decrease in bcl-2 expression, in a dose-dependent manner. Overall, the results suggested that oxymatrine is capable of regulating the expression of apoptosis-associated proteins in prostate cancer cells, *in vitro* and *in vivo*.

In conclusion, the results of the present study demonstrated that oxymatrine exhibits antitumor properties in prostate cancer cells, *in vitro* and *in vivo*. Furthermore, the results suggested that the antitumor properties of oxymatrine may be attributed to the inhibition of proliferation and the induction of apoptosis via regulation of the expression of apoptosis-associated proteins. Therefore, these findings may provide a novel approach for the development of prostate cancer therapy, using oxymatrine, which is derived from the traditional Chinese herb, *Sophora flavescens*.
